# Data on whole genome shotgun sequencing report of clinical *S. maltophilia* strains from India

**DOI:** 10.1016/j.dib.2018.10.005

**Published:** 2018-10-04

**Authors:** Naveen Kumar Devanga Ragupathi, Balaji Veeraraghavan

**Affiliations:** Department of Clinical Microbiology, Christian Medical College, Vellore, Tamilnadu, India

## Abstract

*Stenotrophomonas maltophilia* is an important emerging nosocomial pathogen with broad level multi-drug resistance. There is a lack of genomic information on *S. maltophilia* to understand the antimicrobial resistance (AMR) mechanism behind. The data article reports on whole genome sequence information of 9 clinical *S. maltophilia* strains isolated from a tertiary care hospital in India. Isolates were sequenced using Ion Torrent PGM platform. Raw reads were assembled and annotated, where the genome size ranged from ~ 3.2 to ~ 4.5 Mb with average 57.6× coverage. AMR genes *blaL1*, *blaL2*, *Smqnr*, *aac(6׳)-lz* and *aph(3׳)-llc* were observed among the isolates in addition to multiple virulence factors. Five isolates were identified to be ST15, ST283, ST284, ST285 and ST286.

## Specifications table

**Table:** 

Subject area	*Biology*
More specific subject area	*Microbial genome*
Type of data	*Whole genome shotgun sequences, figure*
How data was acquired	*Ion Torrent PGM*
Data format	*Analyzed*
Experimental factors	*S. maltophilia strains were cultured on blood agar medium. Genomic DNA from cultures were isolated using QIAamp DNA mini kit (Qiagen, Germany).*
Experimental features	*Sequencing was performed according to Ion Torrent PGM specific protocols for library preparation and DNA-seq*.
Data source location	*Mumbai, India, 12.9165° N, 79.1325° E*
Data accessibility	*Data is with this article. Also, genome data are available at GenBank under the accession numbers*
	PXIJ00000000, PXIO00000000, PXIL00000000, PXIN00000000, PXIK00000000, PXII00000000, PXIM00000000, PXJF00000000, PXJG00000000
	https://www.ncbi.nlm.nih.gov/nuccore/PXIJ00000000
	https://www.ncbi.nlm.nih.gov/nuccore/PXIO00000000
	https://www.ncbi.nlm.nih.gov/nuccore/PXIL00000000
	https://www.ncbi.nlm.nih.gov/nuccore/PXIN00000000
	https://www.ncbi.nlm.nih.gov/nuccore/PXIK00000000
	https://www.ncbi.nlm.nih.gov/nuccore/PXII00000000
	https://www.ncbi.nlm.nih.gov/nuccore/PXIM00000000
	https://www.ncbi.nlm.nih.gov/nuccore/PXJF00000000
	https://www.ncbi.nlm.nih.gov/nuccore/PXJG00000000

## Value of the data

• *S. maltophilia* genome data will be useful to understand the genetic make-up of clinical isolates for its associated pathogenicity.
• The genome data will reveal the AMR and virulence profile of *S. maltophilia* from India.
• The data will be helpful for comparison of nosocomial spread *S. maltophilia* from India and to identify the clonal groups.

## 1 Data

**Fig. 1:**
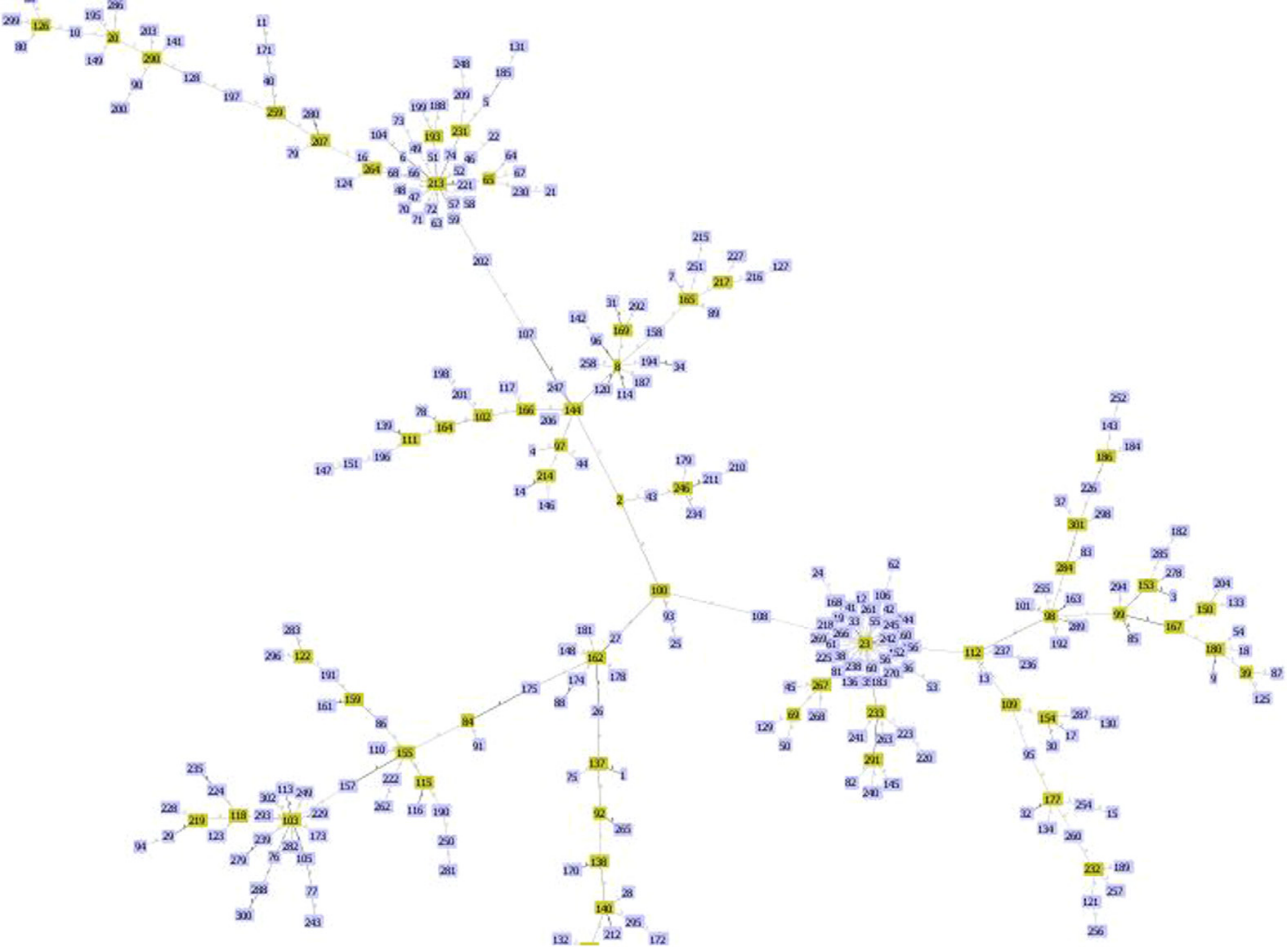
goeBURST analysis of 9 clinical *S. maltophilia* strains in relation to the global strains.

The data presented is on genome sequences of *S. maltophilia* strains from clinical nosocomial infections. The data in Table 1 represents genome annotation summary, including genome size and coverage of each *S. maltophilia* genome. Table 1 also describes the number of tRNA, rRNA, virulence factors from victors and virulence factors database, number of genetic resistance determinants from PATRIC, The Comprehensive Antibiotic Resistance Database and National Database of Antibiotic Resistant Organisms. Table 2 represents various genetic factors responsible for virulence of *S. maltophilia* strains. Multiple antimicrobial resistance (AMR) genes were identified responsible for aminoglycosides, beta-lactams and fluoroquinolones resistance in addition to efflux genes. goeBURST analysis of the study isolates exhibited the clonal relation between the clinical study isolates to the global strains as depicted in Fig. 1.

**Table 1:** Whole genome characteristics of *S. maltophilia* clinical strains (*n* = 9).

**S. no.**	**ID**	**Sequence types**	**Genome size (bp)**	**Coverage (X)**	**CDS**	**tRNA**	**rRNA**	**Victors**	**VFDB**	**PATRIC**	**CARD**	**NDARO**	**Accession no.**
1	S04330	ST286	4,954,343	51.76	5276	72	11	5	1	32	10	3	PXIJ00000000
2	B23119	–	4,507,748	20.16	6919	88	8	13	2	36	24	5	PXIO00000000
3	B27164	ST15	4,568,626	101.02	4875	65	10	4	1	21	16	4	PXIL00000000
4	B26847	ST283	4,582,667	62.18	4838	65	7	4	1	20	14	4	PXIN00000000
5	B09516	–	4,149,004	17.93	5952	63	8	3	1	23	22	3	PXIK00000000
6	S04501	ST284	4,275,498	66.37	4660	77	4	5	2	22	17	5	PXII00000000
7	B26854	–	3,244,183	23.78	5732	60	7	9	4	25	14	3	PXIM00000000
8	B27675	ST285	4,558,790	61.57	4547	80	12	4	1	25	13	3	PXJF00000000
9	B27671	–	4,187,773	17.42	6108	58	7	5	1	34	18	6	PXJG00000000

X – multiples; CDS – coding sequences; VFDB – Virulence Factors Database; CARD – The Comprehensive Antibiotic Resistance Database; NDARO – National Database of Antibiotic Resistant Organisms.

**Table 2:** Virulence and AMR genetic determinants of *S. maltophilia* clinical strains (*n* = 9).

**S. no.**	**ID**	***Smlt***	***afaD***	***hscC***	***RTX***	***smf***	**hemolysin**	**hsp90xo protein**	***pilG***	***FliN***	***cheB***	***acr3***	**Aminoglycosides**	***blaL2***	***Smqnr***	**Efflux genes**	**Sequence types**
1	S04330	−	−	−	−	+	+	+	+	+	+	+	–	*blaL2*	*smqnr28*	*EmrA, EmrB, MdtB, MdtA, MdtC*	ST286
2	B23119	−	−	+	−	+	+	+	+	+	+	+	*aac(6׳)-lz, aph(3׳)-llc*	*blaL2*	*smqnr10*	*EmrA, EmrB, MdtB, MdtA, MdtC*	–
3	B27164	−	−	−	−	+	+	+	+	+	+	+	–	*blaL2*	*smqnr28*	*EmrA, EmrB, MdtB, MdtA, MdtC*	ST15
4	B26847	−	−	−	+	+	+	+	+	+	+	+	*aph(3׳)-llc*	*blaL2, blaL1*	*incomplete qnr*	*EmrA, EmrB, MSF, MdtB, MdtA, MdtC*	ST283
5	B09516	−	−	−	−	+	+	+	+	+	+	+	*aph(3׳)-llc*	*blaL1*	*smqnr42*	*EmrA, EmrB, MdtB, MdtA, MdtC*	–
6	S04501	−	−	−	−	+	+	+	+	+	+	+	*aac(6׳)-lz, aph(3׳)-llc*	*blaL2, blaL1*	*smqnr44*	*EmrA, EmrB, MdtB, MdtC*	ST284
7	B26854	−	−	−	+	+	+	+	+	+	+	+	–	*blaL2*	*smqnr40*	*EmrA, EmrB, MdtB, MdtA, MdtC*	–
8	B27675	−	−	−	+	+	+	+	+	+	+	+	–	*blaL2*	*smqnr35*	*EmrA, EmrB, MdtB, MdtA, MdtC*	ST285
9	B27671	−	−	−	+	+	+	+	+	+	+	+	–	*blaL2*	–	*MdtA, MdtB, MdtC*	–

## 2 Experimental design, materials and methods

### 2.1 Study isolates

*S. maltophilia* clinical strains were isolated from blood and sputum specimens, collected between May 2017 and October 2017 in the Department of Clinical Microbiology, Christian Medical College, Vellore, India.

### 2.2 DNA extraction and genome sequencing

QiAamp DNA mini Kit (Qiagen, Germany) was used to extract the genomic DNA. Ion Torrent PGM platform (Life Technologies) was used for genome sequencing with 400 bp chemistry as per manufacturers’ instructions.

### 2.3 De novo assembly and annotation

Raw reads were assembled de novo in AssemblerSPAdes v.5.0.0.0 embedded in Torrent suite server v.5.0.5. PATRIC database (the bacterial bioinformatics database and analysis resource) (http://www.patricbrc.org) [1], and the NCBI Prokaryotic Genome Automatic Annotation Pipeline (PGAAP) (http://www.ncbi.nlm.nih.gov/genomes/static/Pipeline.html) were used for annotation of the *S. maltophilia* genomes.

The *S. maltophilia* genomes ranged in sizes from ~ 3.2 to ~ 4.5 Mb. The genomes had a coverage ranging from 17× to 153× (Table 1). The Coding DNA sequences (CDS) per genome were between 4547 and 7275, while the tRNA were from 42 to 88, and rRNA from 4 to 12.The number of virulence genes identified as per Victor׳s database were 1–13, and as per VFDB were 1–4. The AMR genes identified ranged from 17 to 36, 8 to 24 and 3 to 7 as per PATRIC, CARD and NDARO databases respectively. The draft genome sequences have been deposited in GenBank under the accession numbers provided in Table 1. The version described in this manuscript is version 1.

AMR genes in *S. maltophilia* genomes were identified using ResFinder 2.1 [2] and plasmids using PlasmidFinder 1.3 [3]. Four isolates (B26847, B09516, S04501, B23119) harboured aminoglycoside resistance genes *aph(3׳)-llc* and two had *aac(6׳)-lz* (B23119, S04501). Three isolates (B26847, B09516, S04501) harboured beta-lactamase gene *blaL1*, whereas *blaL2* was positive in all except B09516. Variants of *Smqnr* was present in all except B27671 (Table 2). None of the isolates harboured any plasmids.

Virulence factors, *smf* (fimbrial adhesion protein), hemolysin, *hsp90* (heat shock protein), *pilG* (twitching motility protein), *FliN* (flagellar motor switch protein), *cheB* (chemotaxis regulator) and *acr3* (arsenical-resistance protein) were present in all 9 isolates (Table 2). *RTX* (repeats-in-toxin) gene was present in B26847, B26854, B27675 and B27671, while *hscC* (chaperone heat shock protein hsp70) was present only in B23119. All isolates were negative for *Smlt* (protein of type IV secretion system) and *afaD* (non-fimbrial adhesion). Genomes were also analysed for the presence of pathogen islands by NCBI-BLAST which resulted negative for all genomes.

MLST 1.8 database was employed to identify the sequence types (STs) (https://cge.cbs.dtu.dk//services/MLST/) [4]. Five isolates were identified with their STs, B27164-ST15, B26847-ST283, S04501-ST284, B27675-ST285 and S04330-ST286. Among other four isolates, allele sequences for *mutM* exhibited < 50% similarity to the available reference sequences in the PubMLST database. goeBURST analysis was performed for the study isolates using PHYLOViZ 2.0 tool [5], which exhibited the relation between the clinical study isolates to the global strains (Fig. 1). The isolates observed in this study are singletons and does not emerge from same ancestor.
